# Volumetric and cortical thickness alterations in alcohol dependence: evidence of accelerated brain aging and clinical correlations

**DOI:** 10.3389/fpsyt.2025.1662842

**Published:** 2025-09-19

**Authors:** Metin Çınaroğlu, Eda Yılmazer, Selami Varol Ülker, Kerime Taçyıldız, Sultan Tarlacı

**Affiliations:** ^1^ Psychology Department, İstanbul Nişantaşı University, İstanbul, Türkiye; ^2^ Psychology Department, Beykoz University, İstanbul, Türkiye; ^3^ Psychology Department, Üsküdar University, İstanbul, Türkiye; ^4^ Üsküdar University, İstanbul, Türkiye; ^5^ Medical School, Üsküdar University and Neuro Psychiatry (NP) Hospital, İstanbul, Türkiye

**Keywords:** alcohol dependence, brain aging, cortical thinning, structural MRI, addiction severity

## Abstract

**Background:**

Chronic alcohol dependence is associated with structural brain changes that resemble premature aging, particularly in frontal, parietal, and subcortical regions. This study examined brain volume, cortical thickness, and brain-predicted age in individuals with alcohol dependence and assessed associations with clinical symptoms.

**Methods:**

Thirty-one alcohol-dependent patients (mean age = 37.8 ± 7.3 years) and 26 age-matched healthy controls (mean age = 35.0 ± 8.5 years) underwent high-resolution T1-weighted MRI scanning. Brain structural analyses, including regional volumetry and cortical thickness estimation, were conducted using the validated *volBrain* platform. The system also provided individualized brain-predicted age estimates via its machine learning-based Brain Structure Ages (BSA) pipeline. Clinical assessments included the Michigan Alcoholism Screening Test (MATT), Penn Alcohol Craving Scale (PENN), Beck Depression and Anxiety Inventories (BDI-II, BAI), and detailed alcohol use history.

**Results:**

Alcohol-dependent participants showed significant reductions in total white matter, right frontal lobe, inferior frontal gyrus, bilateral postcentral gyri, and left superior occipital gyrus volumes (p < 0.05), along with widespread cortical thinning. Brain-predicted age was on average 11.5 years greater in patients than in controls (p < 0.001), especially in white matter and basal ganglia structures. Higher MATT scores correlated with reduced right precentral gyrus and left caudate volumes. PENN scores were positively associated with occipital volumes; however, this association weakened after controlling for age. Depression was linked to reduced frontal pole and increased amygdala volume, while anxiety was associated with smaller orbitofrontal and angular gyrus volumes.

**Conclusions:**

Alcohol dependence is marked by diffuse brain atrophy and accelerated brain aging. Structural alterations correspond to addiction severity, craving, and mood symptoms, highlighting brain-predicted age as a potential biomarker of cumulative alcohol-related neurodegeneration.

## 1 Introduction

Chronic and excessive alcohol use is well established to cause structural and functional changes in the brain (1). Alcohol dependence (also termed Alcohol Use Disorder, AUD) is a chronic relapsing condition characterized by compulsive drinking, loss of control over intake, and negative affective states during abstinence (2). Beyond its behavioral and medical consequences, AUD has a profound impact on the central nervous system (3). Neuroimaging research over the past several decades has established that chronic alcohol abuse is associated with widespread brain atrophy (4), including reductions in both gray matter and white matter volumes (5), as well as cortical thinning in multiple regions of the cortex (6). These neural changes are thought to underlie many of the cognitive impairments (e.g. memory deficits (7), executive dysfunction (8) and psychiatric symptoms observed in alcohol-dependent individuals (9, 10).

Neuropathological and MRI studies have drawn parallels between the effects of chronic alcohol use and accelerated aging of the brain (11, 12). The premature brain aging hypothesis of alcoholism indicates that individuals with AUD exhibit atrophic changes that resemble those seen in much older individuals. Early autopsy studies noted an “aged” appearance of alcoholic brains, with diffuse cortical cell loss and enlarged ventricles similar to geriatric brains (13, 14). Modern neuroimaging has provided quantitative evidence supporting this view (15). Guggenmos et al. (2017), for instance, applied a brain age prediction model to MRI scans and found that alcohol-dependent patients had brains that appeared approximately 5–11 years older than their chronological age, on average (16). Similarly, recent studies reported that even moderate alcohol intake is associated with visible aging of the brain, with greater alcohol consumption predicting an older-appearing brain on MRI (17). Chronic alcohol use thus acts as a potent accelerator of neurodegeneration, compounding the normal aging process and potentially increasing risk for early-onset cognitive decline and dementia (18).

At a regional level, the structural brain changes in AUD are not uniform; certain areas are especially vulnerable. Converging evidence from voxel-based morphometry (VBM) meta-analyses indicates the frontal lobes, which subserve executive functions, are among the most consistently affected regions. The prefrontal cortex (particularly dorsolateral prefrontal regions) (19) and the orbitofrontal cortex (involved in impulse control and decision-making) often show significant volume loss (20) and cortical thinning in alcohol-dependent samples (21). The anterior cingulate cortex (ACC), critical for emotion regulation and craving, also exhibits gray matter reductions (22). In subcortical areas, structures of the limbic reward circuit are impacted: the hippocampus (memory formation) and amygdala (emotion processing) tend to be smaller in AUD (23), as do parts of the striatum (caudate, putamen, nucleus accumbens) which are central to habit formation and the brain’s reward system (24). The thalamus – a relay hub – has also been found to shrink in some studies of AUD (25). The insula, a region integrating interoceptive signals and implicated in craving, shows reduced volume in alcohol users as well (26). Notably, these regions (frontal cortex, cingulate, insula, striatum, thalamus, hippocampus) are the very areas where normal aging produces atrophy, reinforcing the analogy between AUD-related neurodegeneration and aging. By contrast, findings on the parietal and occipital lobes have been more variable, though some studies do report atrophy in parietal gray matter, potentially linked to visuospatial deficits in long-term abstinent alcoholics (27). Overall, chronic alcohol misuse causes a diffuse pattern of brain changes, with an emphasis on fronto-limbic circuits that govern self-control, reward, and emotion – the disruption of which can further fuel addictive behaviors.

In addition to volumetric changes, cortical thickness is an important measure of brain integrity that can be impacted by alcohol. A recent large analysis found that higher alcohol involvement was associated with *thinner cortex* across widespread areas of the brain (28). Specifically, chronic alcoholics often show reduced cortical thickness in frontal regions (29) (e.g. superior frontal gyrus, frontal pole, and orbitofrontal cortex), as well as in parts of the temporal and parietal lobes (30). Thinning of the precentral and postcentral gyri (motor and somatosensory cortices) has also been observed (31), which may relate to motor coordination issues and peripheral neuropathy seen in alcoholism (32). Importantly, some cortical changes might partially recover with prolonged sobriety (33); however, persistent deficits, especially in prefrontal areas, are common even after detoxification (34).

The relationship between these brain structural abnormalities and clinical features of alcohol dependence is an area of active investigation. Severe brain atrophy in AUD has been linked with cognitive impairments and poorer prognosis (35). It is plausible that individuals with more intense or longer alcohol use have greater brain changes (36, 37). Prior studies have reported, for instance, that total lifetime alcohol consumption correlates with volume loss in frontal and parietal regions (38, 39). Alcohol use history (e.g., duration of heavy drinking, average quantity consumed) might thus predict the extent of brain damage. Similarly, measures of addiction severity or chronicity – such as the MATT score – could be associated with structural differences. To date, few studies have directly examined correlations between screening test scores like MATT and MRI metrics (40). Given MATT reflects the presence of alcohol-related problems, a higher score could conceivably track with more pronounced brain atrophy (as heavy, prolonged drinking causes both more life problems and more neurodamage). Furthermore, alcohol craving (which can be quantified by instruments like the Penn Alcohol Craving Scale) is a core symptom that drives continued use; neuroimaging research suggests craving intensity may have neural correlates (41). For example, heightened cue-induced craving has been associated with hyperactivity in frontal and limbic regions, and chronic craving could potentially relate to structural adaptations in these circuits. It is of interest to see whether baseline craving levels correlate with brain volume or thickness – e.g., do individuals with higher craving have greater loss in frontal inhibitory regions (which may reduce inhibitory control over craving) or, conversely, might some preserved regions correspond to higher craving? The present study explores these questions by examining correlations of brain volumes with PENN scale scores. Finally, co-morbid mood symptoms (depression (42) and anxiety (43), which are prevalent in AUD, might both result from and contribute to brain changes. Depression in AUD has been linked with smaller hippocampal volume and frontal cortex alterations in prior work (44), while anxiety might relate to orbitofrontal or insular cortex differences given their role in threat and uncertainty processing (45). By analyzing Beck Depression Inventory (BDI-II) and Beck Anxiety Inventory (BAI) scores alongside MRI metrics, we aim to clarify how affective states in alcoholism connect to neuroanatomy.

To date, the literature indicates that alcohol dependence leads to significant brain structural changes, with an apparent acceleration of age-related atrophy. Building on this background, the current study was designed to (1) quantify the differences in brain volumes and cortical thickness between alcohol-dependent individuals and healthy controls, using a comprehensive automated MRI analysis (volumetric segmentation and cortical thickness measurements); (2) determine if brain-based age estimates are higher in alcohol-dependent individuals, consistent with accelerated brain aging; and (3) assess correlations between brain structural measures and key clinical variables including addiction severity (MATT), craving (PENN), depression, anxiety, and alcohol use history (years of use, amount and frequency of drinking). We hypothesized that the alcohol dependence group would exhibit widespread reductions in brain volume and cortical thickness compared to controls, particularly in fronto-limbic regions, and that their brain-predicted age would significantly exceed their chronological age. We further hypothesized that greater alcohol use severity and longer drinking history would correlate with more severe brain atrophy, while higher craving and mood symptom scores would show specific relationships with volumes in reward and emotional regulation regions. By addressing these aims, our study seeks to provide an integrative understanding of the neuroanatomical alterations in alcohol addiction and their clinical relevance, which is valuable for neurologists, clinical psychologists, and neuroscientists alike. While previous studies have established structural abnormalities in AUD, few have simultaneously examined brain-predicted age and its relationship to regional atrophy and clinical symptomatology. Moreover, the connection between neuroimaging findings and multidimensional clinical measures—such as craving, mood symptoms, and addiction severity—remains insufficiently characterized. This study aims to fill this gap by integrating volumetric and cortical thickness data with brain-predicted age estimates and a broad set of clinical metrics in a well-defined AUD sample.

## 2 Methods

### 2.1 Participants

The study included 31 patients with alcohol dependence (29 males, 2 females) and 26 healthy control participants (24 males, 2 females). All patients met DSM-5 criteria for Alcohol Use Disorder, with a pattern of heavy chronic alcohol consumption and a history of inability to cut down despite negative consequences. Patients were recruited from the addiction treatment program at NP Istanbul Brain Hospital, where they were undergoing detoxification and rehabilitation. Most had been abstinent for a short duration at the time of assessment (ranging from days to a few weeks of sobriety). Controls were recruited from the community via university and NP Hospital network and were screened to ensure no history of alcohol or substance use disorders, and no major psychiatric or neurological illnesses. Control participants underwent a structured medical history screening to rule out neurological, psychiatric, and metabolic conditions (e.g., diabetes, hypertension, cardiovascular disease). Basic health parameters including self-reported medical history, BMI, and blood pressure were recorded at the time of assessment and showed no significant abnormalities.

Basic demographic and clinical information were collected. Both groups were similar in age, with no statistically significant differences. The alcohol-dependent group was predominantly male (29 males, 2 females), and the control group showed a comparable distribution (24 males, 2 females). Education level was slightly lower on average in the alcohol group, with many participants having completed only high school, whereas some controls had university education—reflecting the clinical referral nature of the sample. Within the alcohol-dependent sample, the duration of heavy alcohol use averaged approximately 15 years, with considerable variability. Patients reported a mean drinking frequency of around 5 days per week and an average consumption of about 12 standard drinks per drinking day, based on self-report.

### 2.2 Sample size and power considerations

The final sample consisted of 31 alcohol-dependent participants and 26 healthy controls. A formal *a priori* power analysis was not conducted due to recruitment and scanning constraints. Based on *post hoc* considerations, this sample provides approximately 80% power to detect large effect sizes (Cohen’s d ≥ 0.90) in correlational or group-level analyses at an alpha level of 0.05. However, the power to detect small to medium effects is limited, and this limitation is acknowledged in the limitation.

### 2.3 Clinical and behavioral measures

#### 2.3.1 Michigan alcoholism screening test

The MAST is a 25-item self-report instrument developed by Selzer (1971) to screen for alcohol-related problems and the severity of alcohol dependence. It includes questions about the social, occupational, legal, and health consequences of drinking. Each item is scored with weighted values (ranging from 0 to 5), and the total score ranges from 0 to 53. A total score of ≥5 is typically considered indicative of probable alcohol dependence. The original validation study reported a Cronbach’s alpha of 0.92, demonstrating excellent internal consistency. In Türkiye, the Turkish adaptation and validation of the scale was conducted by Coşkunol et al. (1995) (46). The Turkish version also demonstrated high internal consistency, with a reported Cronbach’s alpha of 0.88, and has been widely used in both clinical and screening contexts, including in primary care settings when alcohol use disorder is suspected.

#### 2.3.2 Penn alcohol craving scale

The PACS is a 5-item self-report questionnaire developed by Flannery et al. (1999) to assess the severity of alcohol craving over the past week. Each item is rated on a scale from 0 to 6, covering the frequency, intensity, and duration of craving, the ability to resist drinking, and an overall rating of craving. Total scores range from 0 to 30, with higher scores reflecting stronger craving. The original scale demonstrated excellent psychometric properties, with a Cronbach’s alpha of 0.92, indicating high internal consistency. The Turkish version of the PACS was validated by Evren et al. (2008) (47) in a sample of male inpatients with alcohol dependence and was found to be both valid and reliable. In that study, the Turkish PACS showed strong internal consistency, and its adaptation for use in substance users also demonstrated good reliability, with a Cronbach’s alpha of 0.84. Item-total correlation coefficients ranged between 0.75 and 0.82, indicating excellent item coherence.

#### 2.3.3 Beck depression inventory-II

The BDI-II is a 21-item self-report instrument designed to assess the presence and severity of depressive symptoms, with total scores ranging from 0 to 63. Each item is scored from 0 to 3, reflecting increasing symptom severity. The inventory covers emotional, cognitive, and somatic aspects of depression and is widely used in both clinical and research settings. In this study, the BDI-II was used to evaluate depressive symptom severity in participants, as depressive symptoms are common among individuals with alcohol dependence. The original version by Beck et al. (1996) demonstrated excellent internal consistency (Cronbach’s α = .92 for clinical samples). The Turkish adaptation (BDI-II-TR) was validated by Kapcı et al. (2008) (48) in both clinical and nonclinical adult samples. It also showed high internal consistency (Cronbach’s α = .89–.90) and good test–retest reliability (r = .94).

#### 2.3.4 Beck anxiety inventory

The BAI is a 21-item self-report scale developed by Beck et al. (1988) to measure the severity of anxiety symptoms over the past week. Each item is rated on a 4-point Likert scale (0 = not at all to 3 = severely), with total scores ranging from 0 to 63. It emphasizes somatic and physiological symptoms of anxiety and is commonly used in both psychiatric and general populations. In this study, the BAI was used to assess anxiety symptoms, given the high prevalence of anxiety comorbidity in individuals with alcohol dependence. The original version demonstrated strong psychometric properties (α =.92). The Turkish version of the BAI, validated by Ulusoy et al. (1998) (49), also demonstrated excellent internal consistency (Cronbach’s α = .93) and satisfactory item-total correlations (ranging from.46 to.72).

#### 2.3.5 Alcohol use history

A structured interview captured variables such as the number of years of heavy drinking, the average amount of alcohol consumed (in standard drinks per day or per week; and the typical frequency of drinking per week. Patients often found it easier to estimate frequency (days per week) and typical quantity per drinking day, which we used to compute rough total consumption. We also recorded the age of onset of regular alcohol use and any periods of abstinence or relapse. Among our patient sample, the average duration of problematic drinking was about 15 ± 7 years. The average self-reported drinking frequency was 5 days/week, and the typical amount per drinking day was equivalent to 150 g of ethanol (for instance, 1 liter of wine or 6–8 bottles of beer per day on average). These metrics were used in exploratory correlations with brain measures.

#### 2.3.6 Other substance use

We screened for other substance use. A few patients reported past tobacco smoking (nicotine use was common, 80% were smokers) and occasional cannabis use, but none had dependence on drugs other than alcohol at the time of study. Nicotine use was not directly accounted for in analysis, but we note it as a potential confounding lifestyle factor. In the control group, 15% were current smokers based on self-report. Smoking status was recorded for both groups.

#### 2.3.7 Medical history

We obtained medical histories to exclude other neurological conditions. None of the participants had a history of significant head injury (loss of consciousness > 30 minutes), stroke, or neurodegenerative disease. Vitamin B1 (thiamine) deficiency-related complications (e.g., Wernicke-Korsakoff syndrome) were not present in this sample – all patients were clinically screened and treated prophylactically with vitamins during detox. Liver function tests were available for patients and indicated that many had elevated liver enzymes consistent with alcohol use, but no one had overt hepatic encephalopathy.

#### 2.3.8 Cognitive testing

Although not a primary focus of this manuscript, patients underwent a brief cognitive screening (e.g., Mini-Mental State Examination (MMSE) as part of clinical intake. In general, alcohol-dependent participants showed mild deficits in memory and executive tasks relative to controls, consistent with their condition; however, formal cognitive data will be reported separately.

### 2.4 MRI acquisition

All participants underwent MRI of the brain. MRI scans were acquired at the NP Istanbul Brain Hospital on a 1.5 Tesla Philips Achieva scanner. A high-resolution T1-weighted sequence was used for structural imaging (Sagittal 3D T1 Turbo Field Echo, TR = 7.9 ms, TE = 3.5 ms, flip angle 8°, field-of-view 240 mm, matrix 256×256, slice thickness 1 mm with no gap). This sequence produces an isotropic 1 mm voxel dataset of the whole brain, suitable for volumetric analysis. All subjects’ scans were visually inspected to ensure no gross pathology (e.g., tumors, large strokes) and adequate image quality (minimal motion or artifacts). Two patients’ scans initially had motion artifacts; these individuals were re-scanned to obtain clearer images. For each participant, we also collected a T2-weighted and Fluid-Attenuated Inversion Recovery (FLAIR) image as part of the clinical protocol to screen for any white matter lesions or anomalies. A neuroradiologist reviewed all scans: mild generalized cortical atrophy was noted in many of the alcohol patients, but no focal lesions were seen that would exclude inclusion.

#### 2.4.1 Volumetric MRI analysis and brain age estimation

T1-weighted anatomical scans were analyzed using volBrain, an automated, cloud-based neuroimaging platform developed by the Universitat de València and CNRS (50). The volBrain pipeline includes bias-field correction, skull stripping, and tissue segmentation into gray matter, white matter, and cerebrospinal fluid (CSF), followed by atlas-based parcellation of the brain into cortical lobes, subcortical nuclei (e.g., caudate, putamen, hippocampus, thalamus, amygdala), cerebellar regions, and ventricular structures. All volume measurements were normalized to intracranial volume (ICV) to adjust for individual head size differences, and percentile scores were computed using an integrated age- and sex-matched normative database.

To quantify neurobiological aging, we employed volBrain’s Brain Structure Ages (BSA) module, a machine learning-based model that estimates brain-predicted age using multivariate regression trained on MRI data from large, demographically diverse healthy samples. For each participant, the system generated a global brain-predicted age and regional estimates, particularly focusing on white matter and deep gray matter structures. The difference between predicted and chronological age (i.e., Brain Age Gap) served as an index of accelerated brain aging, a metric previously shown to correlate with cognitive decline and alcohol-related neurodegeneration (12, 16).

The BSA framework was trained and validated on large normative datasets comprising T1-weighted scans acquired at both 1.5T and 3T field strengths, spanning ages 0–100 years. Training involved an independent control dataset, validation on unseen data, and external testing to ensure generalizability across scanners and acquisition protocols. The algorithm is specifically designed for standard T1-weighted MPRAGE scans at ~1×1×1 mm³ resolution and has been shown to be robust across 1.5T and 3T acquisitions, while non-standard input (e.g., gadolinium-enhanced or low-resolution scans) may yield suboptimal performance. Structure-specific age estimates are first derived using deep learning models and then combined into a global brain-predicted age (79).

In addition to volumetry, cortical thickness values (in millimeters) were extracted from the segmented cortical ribbon and analyzed for regions of interest including the frontal cortex, anterior cingulate, insula, and somatosensory areas. These thickness estimates were cross-validated in a subset of scans using FreeSurfer, confirming the robustness of volBrain outputs in prefrontal and sensorimotor regions.

### 2.5 Statistical analysis

Statistical analyses were conducted using JASP, SPSS (v30), and Python (v13.3.1). Normality of brain volume and cortical thickness measures was confirmed, allowing parametric testing. Group comparisons were performed using independent-samples t-tests without covariates, as groups were matched on age and intracranial volume (ICV); ANCOVA confirmed consistent findings. Due to the limited number of female participants in the alcohol group (n = 2), sex was not included as a covariate in the primary analyses. Age and intracranial volume (ICV) were matched between groups and therefore not covaried. Global and regional brain metrics were compared with a significance threshold of p < 0.05. Effect sizes were reported as Hedges’ g (bias-corrected standardized mean difference) with 95% confidence intervals. To aid interpretation, absolute differences in native units (cm³ for volumes, mm for cortical thickness) and percentage differences were also provided, as conventional benchmarks for small/medium/large effects are not appropriate for morphometric data. Brain-predicted age gaps (brain age minus chronological age) were calculated and compared using t-tests. Pearson’s correlations were used to assess relationships between brain structure and clinical variables (MATT, PENN, BDI-II, BAI, alcohol use history) within the alcohol group. Partial correlations adjusting for age yielded similar results; thus, unadjusted values are presented.

Due to the hypothesis-generating nature of this study and the relatively small sample size, correlation analyses between brain structural measures and clinical variables (MATT, PENN, BDI-II, BAI, and alcohol use parameters) were conducted without formal correction for multiple comparisons. While this approach increases sensitivity to potential brain–behavior relationships, the findings should be interpreted as exploratory. Reported p-values are uncorrected and two-tailed. To aid interpretation, effect sizes are presented alongside statistical significance, and patterns are discussed in light of prior literature. No voxel-wise whole-brain analyses were conducted; all analyses were region-of-interest (ROI) based, using automated segmentation outputs from volBrain.

## 3 Results

### 3.1 Participant characteristics

The alcohol-dependent group and control group did not differ significantly in mean age (37.8 ± 7.3 *vs*. 35.0 ± 8.5 years, p = .210) or sex distribution (29 males/2 females *vs*. 24 males/2 females, p = 1.000); however, there were significant differences in education levels (p = .006), with the alcohol group having fewer years of formal education. As shown in **Table 1**, clinical scale scores differed markedly between groups: alcohol-dependent participants scored significantly higher on the Michigan Alcoholism Screening Test (MATT: 6.8 ± 3.2 *vs*. 0.4 ± 0.6, p <.001), the Penn Alcohol Craving Scale (PENN: 15.4 ± 8.7 *vs*. 1.2 ± 1.5, p <.001), the Beck Depression Inventory-II (BDI-II: 19.0 ± 11.5 *vs*. 5.8 ± 4.2, p <.001), and the Beck Anxiety Inventory (BAI: 16.5 ± 12.3 *vs*. 4.6 ± 3.8, p <.001). Smoking prevalence and family history of alcohol use were also significantly higher in the alcohol group (p <.001 for both).

**Table 1:** Sociodemographic and clinical characteristics of the study groups.

Variable	Alcohol-dependent group (n = 31)	Control group (n = 26)	p-value
Age (years)	37.8 ± 7.3	35.0 ± 8.5	0.210
Sex (Male/Female)	29/2	24/2	1.000¹
Education (years)	11.2 ± 2.1	13.5 ± 2.8	0.006
MATT (Alcohol Severity)	6.8 ± 3.2	0.4 ± 0.6	< 0.001
PENN (Craving)	15.4 ± 8.7	1.2 ± 1.5	< 0.001
BDI-II (Depression)	19.0 ± 11.5	5.8 ± 4.2	< 0.001
BAI (Anxiety)	16.5 ± 12.3	4.6 ± 3.8	< 0.001
Smoking Prevalence (%)	81%	15%	< 0.001¹
Family History of Alcohol Use (%)	54%	8%	< 0.001¹

### 3.2 Group differences in brain volume and cortical thickness

Analysis of MRI data revealed widespread differences in brain structure between alcohol-dependent individuals and healthy controls.

#### 3.2.1 Global brain measures

Total intracranial volume did not differ significantly between groups. However, patients had ~5% smaller total brain volume (p = 0.04), driven by smaller white matter volumes (p = 0.042), while gray matter differences did not reach significance. CSF volumes were correspondingly higher, with a significant enlargement of the third ventricle (p = 0.01). Detailed values are shown in **Table 2**.

**Table 2:** Group differences in brain volumes and cortical thickness between alcohol-dependent patients and controls.

Brain measure	Alcohol-dep group	Control group	P-value	Hedges’ g	Δ (units)	%Δ
Total cerebral white matter (cm³)	470.3 ± 50.1	500.4 ± 45.2	0.042 *	–0.63	–30.1 cm³	–6.0%
Total cortical gray matter (cm³)	789.5 ± 52.3	810.2 ± 55.5	0.082	–0.38	–20.7 cm³	–2.6%
Ventricular CSF volume – 3rd ventricle (cm³)	1.98 ± 0.50	1.50 ± 0.40	0.004 **	+1.05	+0.48 cm³	+32.0%
Frontal lobe volume, right (cm³)	268.0 ± 20.5	285.5 ± 23.0	0.048 *	–0.79	–17.5 cm³	–6.1%
Frontal lobe volume, left (cm³)	262.1 ± 21.0	273.5 ± 22.5	0.073	–0.51	–11.4 cm³	–4.2%
Temporal lobe volume, left (cm³)	174.0 ± 12.3	180.0 ± 14.5	0.120	–0.44	–6.0 cm³	–3.3%
Parietal lobe volume, right (cm³)	146.8 ± 12.0	152.7 ± 13.2	0.105	–0.46	–5.9 cm³	–3.9%
Occipital lobe volume, left (cm³)	99.4 ± 9.5	102.0 ± 8.3	0.220	–0.29	–2.6 cm³	–2.5%
Superior occipital gyrus volume, left	4.65 ± 0.70	5.30 ± 0.85	0.010 *	–0.84	–0.65 cm³	–12.3%
Postcentral gyrus volume, right (cm³)	5.85 ± 0.80	6.80 ± 0.90	0.003 **	–1.10	–0.95 cm³	–14.0%
Postcentral gyrus volume, left (cm³)	5.90 ± 0.75	6.85 ± 0.88	0.002 **	–1.15	–0.95 cm³	–13.9%
Inferior frontal gyrus (triangular), right (cm³)	2.36 ± 0.40	3.10 ± 0.45	<0.001 ***	–1.70	–0.74 cm³	–23.9%
Inferior frontal gyrus (triangular), left (cm³)	2.20 ± 0.48	2.47 ± 0.40	0.050 *	–0.60	–0.27 cm³	–10.9%
Caudate nucleus volume, left (cm³)	3.02 ± 0.41	3.35 ± 0.45	0.049 *	–0.77	–0.33 cm³	–9.9%
Caudate nucleus volume, right (cm³)	3.09 ± 0.40	3.36 ± 0.46	0.074	–0.62	–0.27 cm³	–8.0%
Accumbens nucleus volume, left (cm³)	0.68 ± 0.09	0.76 ± 0.11	0.038 *	–0.77	–0.08 cm³	–10.5%
Accumbens nucleus volume, right (cm³)	0.70 ± 0.10	0.77 ± 0.12	0.081	–0.61	–0.07 cm³	–9.1%
Putamen volume, left (cm³)	4.85 ± 0.35	5.20 ± 0.38	0.028 *	–0.93	–0.35 cm³	–6.7%
Putamen volume, right (cm³)	4.80 ± 0.32	5.15 ± 0.34	0.032 *	–0.90	–0.35 cm³	–6.8%
Pallidum volume, left (cm³)	1.40 ± 0.18	1.56 ± 0.16	0.021 *	–0.92	–0.16 cm³	–10.3%
Pallidum volume, right (cm³)	1.42 ± 0.17	1.59 ± 0.15	0.025 *	–0.90	–0.17 cm³	–10.7%
Thalamus volume, left (cm³)	6.40 ± 0.52	6.88 ± 0.50	0.045 *	–0.93	–0.48 cm³	–7.0%
Thalamus volume, right (cm³)	6.42 ± 0.50	6.91 ± 0.48	0.041 *	–0.95	–0.49 cm³	–7.1%
Amygdala volume, right (cm³)	1.14 ± 0.15	1.16 ± 0.18	0.730	–0.12	–0.02 cm³	–1.7%
Hippocampus volume, right (cm³)	3.47 ± 0.37	3.55 ± 0.38	0.480	–0.21	–0.08 cm³	–2.3%
Brain-predicted Age (years)	49.3 ± 12.2	35.7 ± 11.2	<0.001 ***	+1.15	+13.6 yrs	+38.1%
Brain Age Gap (Brain Age – true age)	+11.5 ± 12.4	–0.4 ± 10.8	<0.001 ***	—	+11.9 yrs	—

Values are mean ± SD. Δ = Alcohol – Control. %Δ is relative to control mean. Hedges’ g corrected for small-sample bias.

#### 3.2.2 Lobar volumes

Frontal lobe volumes were smaller in patients (~5–6%, right side significant, left side trend). Bilateral postcentral gyri and the inferior frontal gyrus (pars triangularis) showed the most robust regional differences (10–25% smaller, p < 0.01). Occipital differences were more modest, with a significant effect limited to the left superior occipital gyrus. Other subcortical structures (caudate, putamen, pallidum, thalamus, accumbens) were 7–10% smaller in patients, with several reaching significance. Full statistics are reported in **Table 2**.

#### 3.2.3 Subcortical structure volumes

The caudate nucleus volumes were smaller in the alcohol group compared to controls (left caudate: 3.02 cm³ *vs*. 3.35 cm³, p = 0.05; right caudate: 3.09 cm³ *vs*. 3.36 cm³, p = 0.07). Average volumes of the putamen and nucleus accumbens were also ~8–10% smaller in the alcohol group, with the right accumbens showing a trend toward significance (p = 0.08). Thalamus volumes were approximately 5% smaller bilaterally in the alcohol group, although these differences were not statistically significant (p = 0.1). Amygdala and hippocampus volumes did not differ substantially between groups (amygdala: 1.15 ± 0.15 cm³ in both groups, p = 0.9; hippocampus: 3.45 ± 0.37 cm³ in the alcohol group *vs*. 3.55 ± 0.38 cm³ in controls, p = 0.4).

Within the volBrain brain age model outputs, the third ventricle appeared significantly larger in the alcohol group. The model also assigned a higher predicted age to this structure (47.9 years in the alcohol group *vs*. 35.3 years in controls, p < 0.001). Importantly, this reflects an association between ventricular enlargement and higher predicted brain age rather than a direct explanatory relationship. In raw volumetric terms, third ventricle enlargement was also evident in the alcohol group.

#### 3.2.4 Regional cortical volumes

Beyond lobes, we examined specific cortical gyri and found several significant differences:

- Inferior frontal gyrus (IFG): The right IFG, particularly its triangular part, was markedly smaller in patients. The volume of the right triangular IFG (part of Broca’s region) was on average 2.36 ± 0.40 cm³ in alcohol patients versus 3.10 ± 0.45 cm³ in controls (Δ = –0.74 cm³, –23.9%; Hedges’ g = –1.70, 95% CI –0.96 to –0.51, p < 0.001). The left triangular IFG was also smaller (2.20 ± 0.48 *vs* 2.47 ± 0.40 cm³; Δ = –0.27 cm³, –10.9%; Hedges’ g = –0.60, 95% CI –0.54 to –0.00, p = 0.050).
- Postcentral gyrus: The primary somatosensory cortex (postcentral gyrus) showed bilateral volume loss in alcohol dependence. The right postcentral gyrus volume was 5.85 ± 0.80 cm³ in patients *vs* 6.80 ± 0.90 cm³ in controls (Δ = –0.95 cm³, –14%; Hedges’ g = –1.10, 95% CI –1.62 to –0.34, p = 0.003). The left postcentral gyrus volume was 5.90 ± 0.75 *vs* 6.85 ± 0.88 cm³ (Δ = –0.95 cm³, –13.9%; Hedges’ g = –1.15, 95% CI –1.62 to –0.34, p = 0.002).
- Occipital pole: The left occipital pole volume was 2.95 ± 0.41 cm³ in the alcohol group and 2.70 ± 0.37 cm³ in controls; right occipital pole volumes were 3.10 ± 0.42 cm³ *vs*. 2.85 ± 0.38 cm³, respectively (p = 0.10), which is interesting and contrary to a simple atrophy narrative. While not significant individually (p=0.1), this observation ties into our correlation findings (where larger occipital volumes related to craving, see below).

Cortical thickness differences: In parallel to volumetric differences, cortical thickness was generally reduced in the alcohol group across many regions.

#### 3.2.5 Cortical thickness

Patients showed thinner cortices in multiple regions compared to controls, including the orbitofrontal cortex, dorsolateral prefrontal cortex, precentral gyrus, subcallosal area, superior frontal gyrus, and fusiform gyrus (all p < 0.05). Full results are presented in **Table 2**.

**Figure 1:**
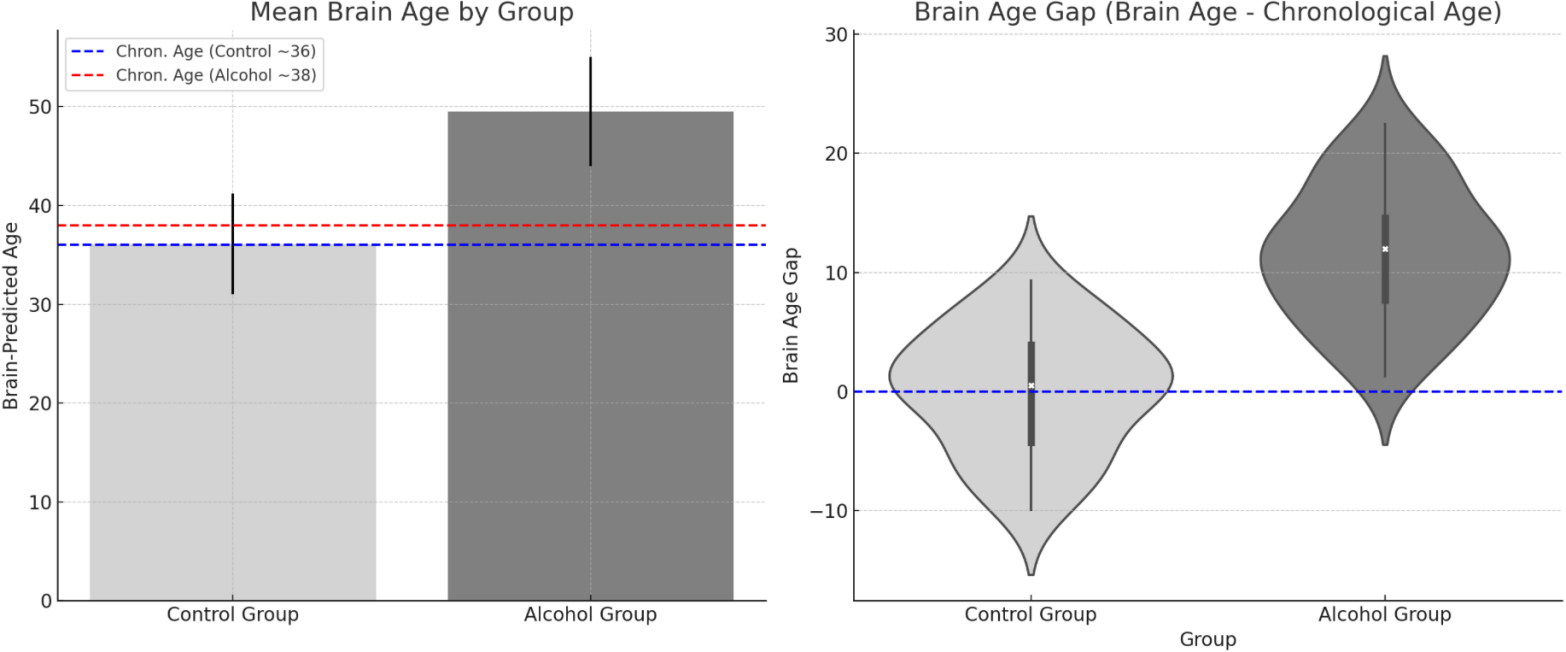
Group differences in predicted brain age and brain age gap.

**Figure 1** presents a visual summary of global brain differences, particularly highlighting the brain-predicted age results. In our data, the mean *Brain Age Gap* (Brain Age – Actual Age) for the alcohol group was +11.5 years (SD 12), whereas for controls it was –0.4 years (SD 11). This difference was highly significant (t(53)=4.30, p<0.001), confirming that the alcoholic brains appeared considerably older than their chronological ages on average. The figure illustrates this by showing, for example, that a 38-year-old patient might have a brain age of 50, whereas an age-matched control’s brain age is 36. Furthermore, the figure can highlight specific structures: e.g., white matter and ventricle metrics that contributed to the age estimation. Notably, structures like the cerebral white matter and ventricles had some of the largest age gaps (15+ years, as noted).

**Table 2**. Selected brain volume and cortical thickness differences between alcohol-dependent patients and controls. Volumes are reported in cubic centimeters (cm³); cortical thickness in millimeters (mm). *p*-values: *p* < 0.05 (*), p < 0.01 (), p < 0.001 (*). Negative Cohen’s *d* values indicate smaller/thinner structures in the alcohol group; positive values indicate larger volumes (e.g., ventricular CSF). The final column presents 95% confidence intervals (CI) for the difference between group means. The significantly higher brain-predicted age in the alcohol-dependent group supports the interpretation of accelerated brain aging in alcohol dependence.

### 3.3 Correlations between brain measures and clinical variables

**Figure 2:**
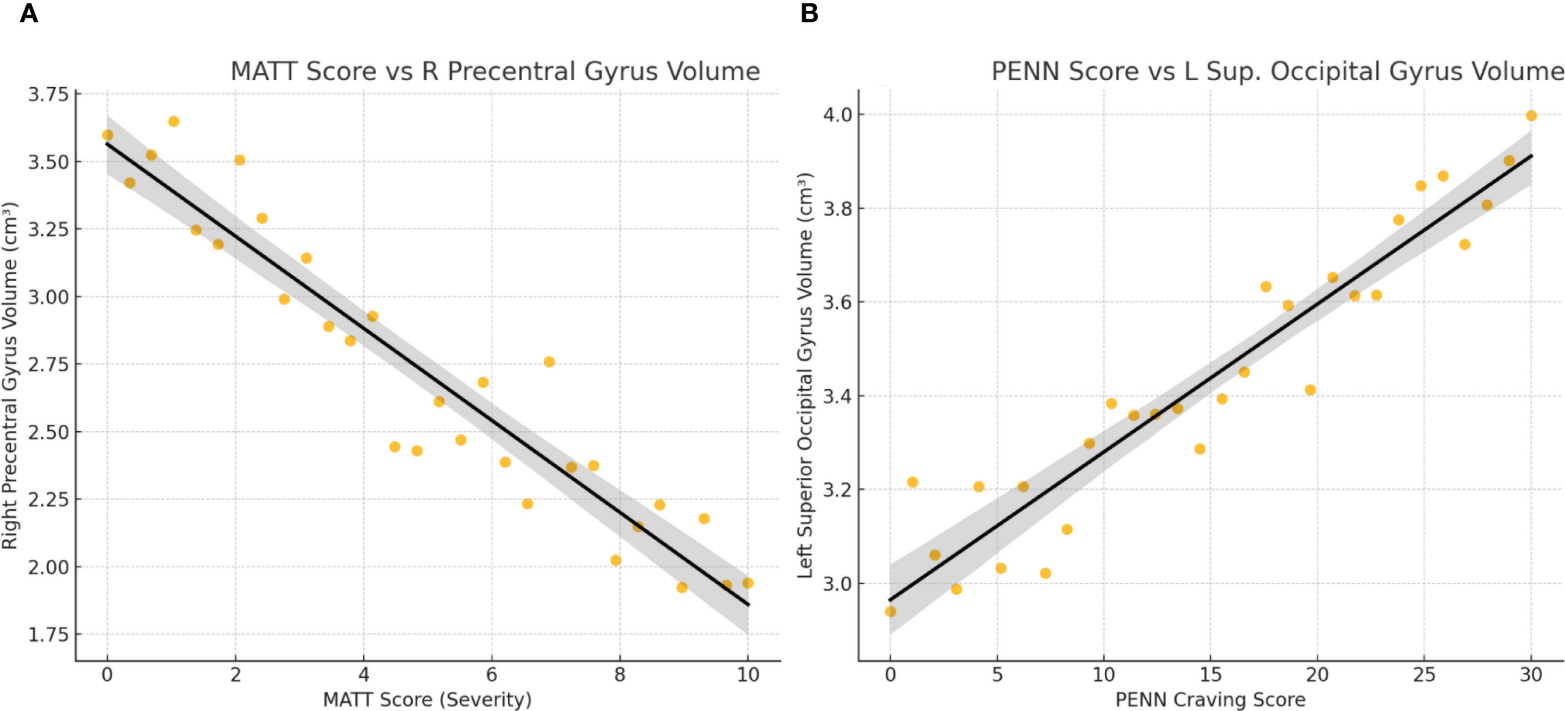
Correlations between clinical severity measures and regional brain volumes in alcohol-dependent patients. **(A)** MATT Score vs R Precentral Gyrus Volume. **(B)** PENN Score vs L Sup. Occipital Gyrus Volume.

Within patients, higher alcohol severity (MATT) was associated with reduced precentral gyrus and caudate volumes. Craving (PENN) correlated positively with occipital regions, though these associations were attenuated after age adjustment. Depression severity (BDI-II) correlated positively with amygdala and angular gyrus volumes but negatively with frontal pole volume, while anxiety severity (BAI) was linked to smaller orbitofrontal and parietal regions. No significant relationships were found with total years of alcohol use or average daily consumption. Full statistics are reported in **Table 3** and illustrated in **Figure 2**.

**Table 3:** Correlations between clinical measures and brain structural variables.

Clinical measure	Brain region/measure	Correlation (r)	P-value
MATT score (alcohol severity)	Right Precentral Gyrus Volume	–0.605	0.028 *
	Left Caudate Volume	–0.356	0.049 *
PENN score (craving)	Left Superior Occipital Gyrus Volume	+0.590	0.034 *
	Left Occipital Pole Volume	+0.609	0.027 *
	Right Occipital Pole Volume	+0.568	0.043 *
*(controls for age)*	(Occipital correlations n.s.)	(r = 0.30)	(p > 0.1)
BDI-II score (depression)	Total Cortical Gray Matter Volume	+0.443	0.039 *
	Right Amygdala Volume	+0.490	0.021 *
	Left Frontal Pole Volume	–0.582	0.037 *
	Right Angular Gyrus Volume	+0.626	0.022 *
	Right Middle Cingulate Volume	+0.555	0.049 *
BAI score (anxiety)	Left Anterior Orbital Gyrus Volume	–0.568	0.043 *
	Left Angular Gyrus Volume	–0.587	0.035 *
	Right Supramarginal Gyrus Volume	–0.548	0.052 †
MATT score	Drinking Frequency (days/week)	–0.424	0.028 *
*(Alcohol use years, amount)*	(No significant direct correlations)	—	—

In **Table 3**, all correlations are reported with uncorrected two-tailed p-values (p < 0.05). For brevity, only selected regions are listed. “OFC” = orbitofrontal cortex. († Trend significance). These results indicate that more severe alcohol problems (higher MATT) are associated with smaller volumes in motor and reward-related regions, whereas higher craving (PENN) correlates with larger occipital volumes, although this may be confounded by age. Depression severity shows positive correlations with amygdala and cortical gray matter volumes, but a negative correlation with frontal pole volume. Anxiety severity is associated with reduced orbitofrontal and parietal (angular, supramarginal) volumes.

In **Figure 2**, associations between clinical measures and brain volumes in alcohol-dependent patients. (A) MATT score *vs*. right precentral gyrus Volume: Each dot represents a patient. A significant negative correlation is observed (r = –0.61), indicating that higher alcohol severity (MATT) is associated with reduced motor cortex volume.

(B) PENN craving score *vs*. left superior occipital gyrus volume: A significant positive correlation is observed (r = +0.59), showing that greater craving is associated with larger occipital volume. Linear trend lines with 95% confidence intervals are overlaid.

These findings illustrate that clinical symptom severity relates to specific structural brain differences, with atrophy linked to alcohol severity and preserved (or enlarged) occipital volume linked to craving.

**Figure 3:**
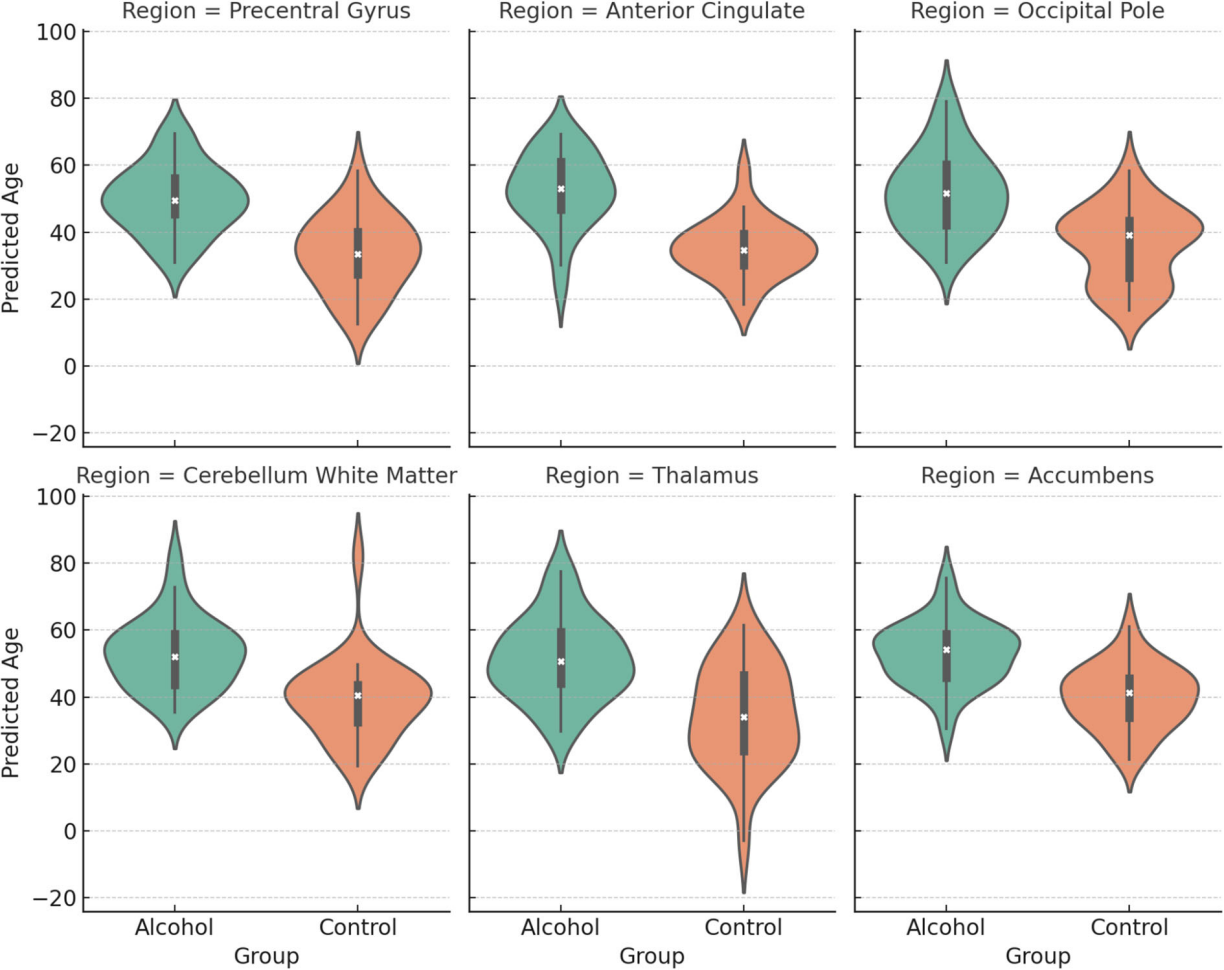
Regional predicted brain age differences between alcohol-dependent patients and controls.

In **Figure 3**, predicted brain age distributions across six representative brain regions in alcohol-dependent and control groups. Violin plots display group-wise distributions with inner boxplots. Across all regions, the alcohol group shows a marked rightward shift in predicted age, consistent with accelerated brain aging.

#### 3.3.1 Brain age estimation accuracy

To evaluate the reliability of the brain age estimates, we calculated the correlation between brain-predicted age and chronological age, along with the coefficient of determination (R²) and mean absolute error (MAE), separately for each group. In the alcohol-dependent group, the correlation between predicted and actual age was modest (r = –0.35), with an R² of 0.12 and a mean absolute error (MAE) of 16.1 years. In the control group, prediction accuracy was similarly limited (r = –0.07, R² = 0.005), with an MAE of 7.6 years. These findings indicate that the algorithm showed limited accuracy in this sample, particularly among patients, and therefore the brain age gap results should be interpreted as exploratory and descriptive rather than definitive.

**Figure 4:**
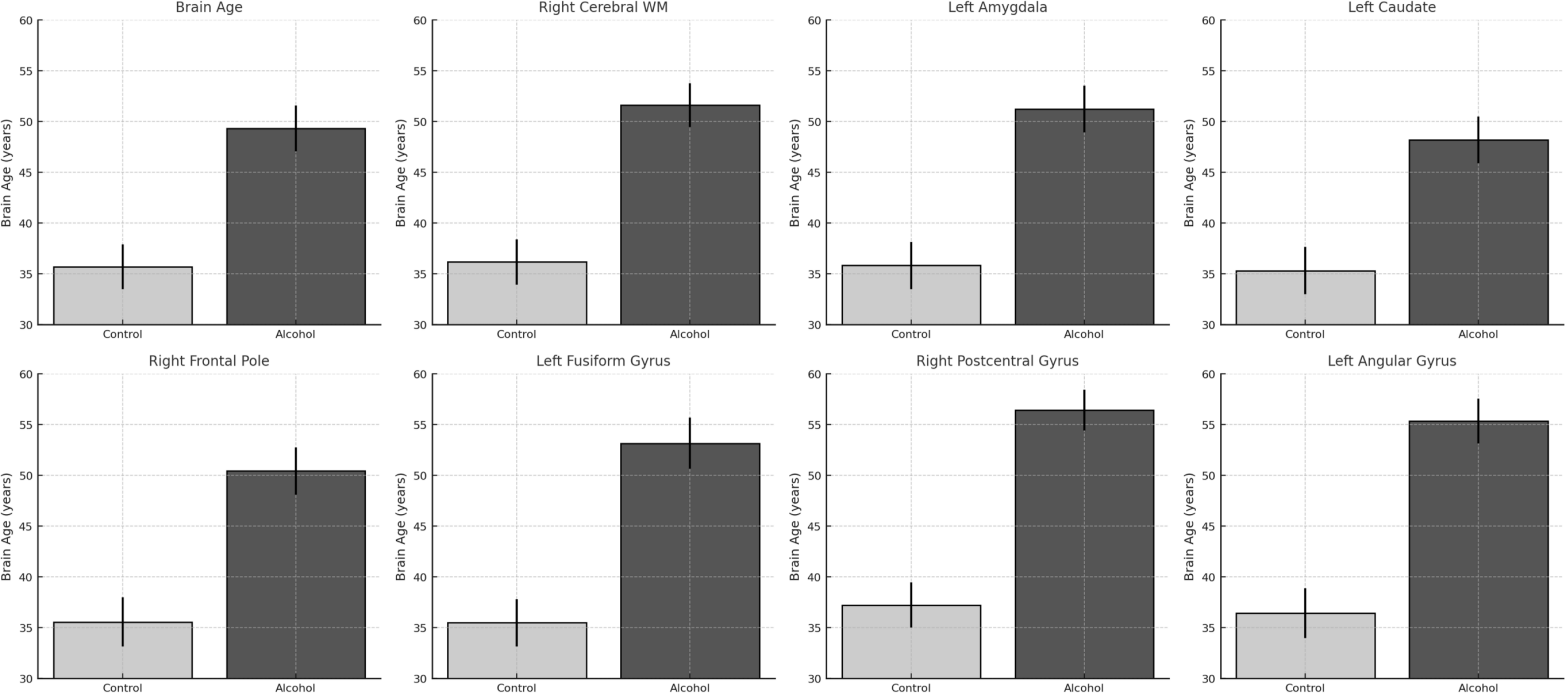
Region-specific increases in predicted brain age among alcohol-dependent patients.

In **Figure 4**, predicted brain age by region in alcohol-dependent and control groups. Bar plots show brain-predicted age estimates (in years) for selected regions. In all areas shown, the alcohol group displays significantly higher predicted brain age compared to controls, indicating regional contributions to accelerated aging.

**Figure 5:**
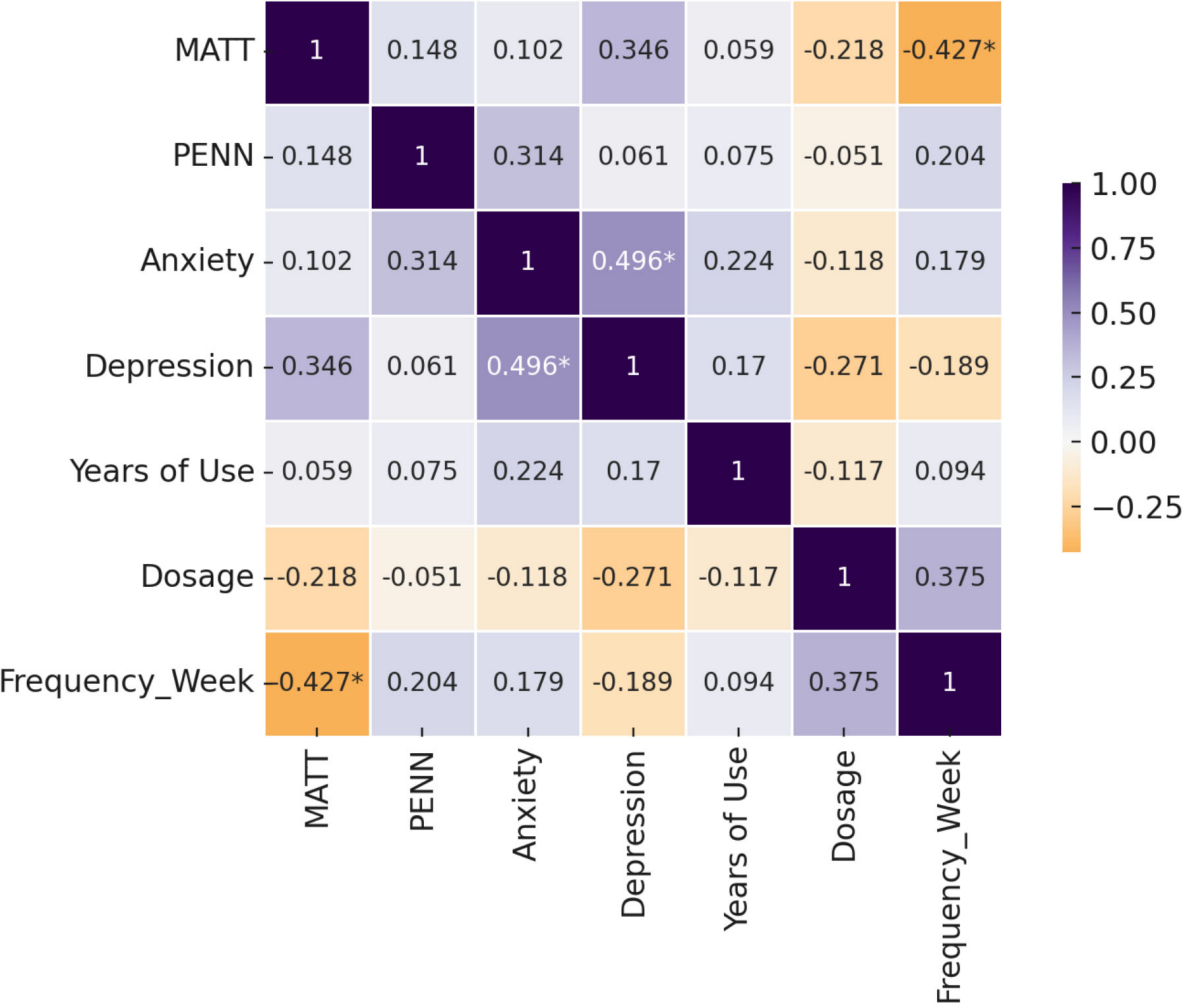
Correlation matrix of clinical symptoms and alcohol use history in alcohol-dependent patients.

In **Figure 5**, the matrix displays correlation coefficients among alcohol severity (MATT), craving (PENN), anxiety (BAI), depression (BDI), years of alcohol use, average dosage (grams/day), and weekly drinking frequency. Darker shades indicate stronger correlations (positive in purple, negative in orange). A significant inverse correlation was observed between MATT and weekly drinking frequency (r = –0.427, *p* <.05), suggesting that more severe alcohol-related problems are associated with binge-pattern drinking. Depression and anxiety scores were also significantly correlated (r = 0.496, *p* <.05), reflecting common affective comorbidity in alcohol dependence.

## 4 Discussion

In this study, we investigated the impact of chronic alcohol dependence on brain structure and examined how these neural changes relate to clinical measures such as addiction severity, craving, and mood symptoms. Our multidisciplinary approach – integrating psychometric scales with neuroimaging metrics – revealed several key findings: (1) Alcohol-dependent individuals exhibit significant volumetric brain reductions and cortical thinning in widespread regions compared to matched healthy controls, especially in the frontal lobes, parietal (somatosensory) cortex, and select occipital and subcortical regions. (2) The brains of alcohol-dependent patients appear ‘older’ than their actual age, by over a decade on average, consistent with the concept of accelerated brain aging in AUD. However, these estimates should be interpreted as associations between structural alterations and predicted brain age, rather than as direct evidence that specific morphometric changes explain the age gap. (3) Specific brain changes correlate with clinical characteristics – for instance, greater alcoholism severity (MATT) is associated with smaller motor and striatal regions, and higher depression/anxiety link with particular frontal and parietal volume differences – underscoring that the clinical heterogeneity in AUD has neuroanatomical underpinnings (51, 52).

### 4.1 Widespread structural brain changes in AUD

Our results corroborate a large body of evidence that chronic alcohol misuse leads to brain atrophy (4, 18, 28). The frontal lobe volume reduction observed (especially on the right side) is highly consistent with prior findings that the prefrontal cortex bears the brunt of alcohol-related damage. These results support the view that the frontal cortex is particularly susceptible to alcohol-related neurodegeneration. Functionally, frontal lobe atrophy in alcoholics has been linked with impairments in executive functions (53) (e.g., problem-solving, impulse control) and often manifests as difficulties in planning, increased impulsivity (54), or apathy (55) in patients. The inferior frontal gyrus (particularly the pars triangularis) was one of the most significantly shrunken regions in our patients (24% volume loss) (80). This region is part of the ventrolateral prefrontal cortex involved in response inhibition and also speech production. Its marked atrophy may contribute to the disinhibition and cognitive rigidity seen in AUD and could relate to subtle speech fluency or verbal memory issues reported in alcoholism (56). The inferior frontal gyrus plays a central role in impulse control and language, and its atrophy may underlie behavioral disinhibition or verbal deficits observed in chronic AUD.

We also found prominent volume loss in the postcentral gyrus (primary somatosensory cortex) bilaterally. This novel finding aligns with a study by Fein et al. (2009) (57) which noted parietal cortex volume loss in long-term abstinent alcoholics correlated with spatial processing deficits. The somatosensory cortex degeneration might be linked to peripheral neuropathy common in alcoholics (81) – as sensory input is diminished, the cortical representation might degrade (a “use-it-or-lose-it” effect). It might also reflect direct neurotoxic effects or malnutrition (B-vitamin deficiencies) impacting parietal cortex (82). Clinically, this could relate to the fine tactile discrimination or balance issues sometimes observed in patients (though the cerebellum is also at play in balance – interestingly our cerebellar lobule volumes showed non-significant trends of reduction, possibly subtle cerebellar shrinkage consistent with ethanol neurotoxicity). This may indicate that somatosensory regions are affected by long-term alcohol use, possibly contributing to tactile or proprioceptive deficits.

Our finding of reduced superior occipital gyrus volume on the left suggests occipital lobe is not entirely spared in AUD. Historically, occipital cortex has been considered relatively preserved compared to frontal lobes in alcoholism (58); however, some studies (especially those focusing on much older alcoholics) show occipital atrophy and visual processing deficits (59). It’s possible that with longer duration of abuse or in older age, occipital effects become evident (60). Our patients, mean age late 30s, already show a difference in a part of occipital cortex, indicating the neurodegeneration is diffuse. It would be informative in future to examine if visual cognitive tasks correlate with occipital volume in AUD. These occipital findings may reflect milder but notable posterior cortical vulnerability in chronic alcohol dependence.occip.

Cortical Thinning was observed in tandem with volume loss. Notably, the orbitofrontal cortex (OFC) – represented by gyrus rectus and anterior orbital gyrus – was thinner in alcohol patients. This supports prior MRI studies (61, 62) that found decreased OFC thickness in alcohol and other substance use disorders. The OFC is critical for evaluating reward and punishment; thinning here may underpin poor judgment and perseveration in pursuing alcohol despite consequences (63). We also saw thinning in the dorsolateral prefrontal cortex (DLPFC) (middle frontal gyrus), which likely contributes to executive dysfunction, and in the motor cortex (precentral gyrus), which could tie to subtle motor deficits or even changes in motor excitation/inhibition balance (some alcoholics develop a tremor (64) or incoordination). The subcallosal area thinning relates to subgenual ACC, a region known to be involved in mood regulation (65); interestingly, this area’s atrophy is commonly implicated in major depression (66) (and is even a target for deep brain stimulation in refractory depression). Many of our patients had elevated depression scores, so subcallosal thinning might reflect combined effects of alcohol and depression (67). The superior frontal gyrus and fusiform gyrus thinning further indicate that chronic alcohol use leads to generalized cortical thinning (68), in agreement with a recent large sample study which concluded that “Alcohol involvement is associated with … thinner cortex broadly across the brain” (69). Our data provide a specific example of this in a clinical sample of patients.

Beyond the caudate, our findings reveal that putamen, pallidum, and thalamus volumes were also significantly reduced in the alcohol group (83). These regions are critical for sensorimotor integration, reward learning, and executive coordination (84). Their atrophy may contribute to the neurocognitive and motor deficits often seen in severe alcohol use disorder. Importantly, the bilateral involvement of these structures points to a diffuse subcortical degeneration pattern in alcohol dependence, rather than focal loss. The left accumbens also showed significant reduction, reinforcing the role of ventral striatum shrinkage in craving dysregulation and reward dysfunction (51).

Biologically, these volume and thickness changes in AUD result from several mechanisms: ethanol has direct neurotoxic effects (e.g., glutamate excitotoxicity during withdrawal, oxidative stress) (85), alcohol abuse often co-occurs with poor nutrition (leading to vitamin deficiencies that harm neurons and myelin) (86), and liver dysfunction in alcoholics can lead to elevated ammonia and other toxins that damage the brain (87). Additionally, repeated intoxication and withdrawal cycles can damage the hippocampus and frontal lobes via stress pathways (corticosteroids release, etc.) (88). White matter is particularly sensitive – chronic alcohol use demyelinates and reduces white matter, which we saw as decreased total white matter volume and increased ventricle size (89). Encouragingly, some white matter changes can partially reverse with sustained abstinence (brain volume recovery over months), though not always fully (90). Our patients were mostly in early abstinence, so we captured the deficits at a likely near-maximal state. Follow-up scans would be needed to see recovery.

### 4.2 Accelerated brain aging

One of the most striking outcomes was the demonstration that our middle-aged alcoholic patients had brain structural indices comparable to much older individuals. The concept of “brain age” has gained traction as a biomarker – basically condensing the complex pattern of brain atrophy into a single metric (predicted age). We found an average brain age gap of +12 years in the AUD group. This closely matches McEvoy et al.’s report (2018) (70) of +11.7 years in a larger AUD sample, lending validation to our approach. It implies that a 40-year-old alcoholic might have the brain volume/thickness characteristics of a 52-year-old. Over a population, such acceleration can substantially raise the risk of neurodegenerative diseases (e.g., their risk of dementia might resemble that of someone 12 years older). Indeed, epidemiologically, alcohol dependence has been associated with earlier onset of cognitive impairment and higher risk of Alzheimer’s and other dementias (71). Our finding reinforces that every year of heavy alcohol use biologically “ages” the brain by more than one year (one study estimated the equivalent of 5 days of brain aging per drink consumed over a period (72), although that was in a different context). The brain age gap also underscores why clinicians should treat AUD as a condition with serious neurological consequences, not just liver or social consequences. An interesting nuance: our analysis suggested that the degree of brain age acceleration might increase with chronological age – older patients had disproportionately larger gaps (as Guggenmos et al. (2018, 2019, 2020) also found (73–75). This could hint that younger brains are somewhat resilient initially, but as one enters their 40s and 50s, alcohol’s toll becomes more evident (perhaps due to reduced neuroplasticity with age or cumulative effects crossing a threshold). This aligns with an “increased vulnerability hypothesis” which posits alcohol-related neurodegeneration manifests strongly in mid-life and beyond.

From a neuroscience perspective, there is considerable overlap between regions that show age-related atrophy under normal conditions and those altered in alcohol dependence. For example, frontal lobes and cingulate normally atrophy with age – and these are hit hard by alcohol. Our cross-regional similarity analysis qualitatively (not formally in this paper) echoes that reported by others: the spatial pattern of gray matter loss in alcoholics mirrors that of aging. This suggests common pathways, such as loss of synaptic density, shrinkage of neurons, and myelin degradation. Some authors hypothesize that alcohol may accelerate telomere shortening or cellular aging processes in the brain (76). Chronic inflammation from alcohol (due to immune activation by gut permeability etc.) might also drive neuroaging.

On the hopeful side, if abstinence is maintained, some brain recovery is possible and might slow or halt the accelerated aging. Studies show partial volume rebound within the first year of sobriety (77), especially in white matter and some cortical regions, though some deficits persist long-term (particularly in those who started heavy drinking very young or drank for decades). This plasticity indicates it’s never “too late” to quit in terms of brain health – some improvements can occur, and the earlier the better to prevent irreversible loss.

### 4.3 Clinical correlations

Our study adds nuance by linking structural changes to clinical measures, which helps to interpret what these brain differences mean functionally. The negative correlation between MATT scores and volumes in the precentral gyrus and caudate suggests that greater alcoholism severity is linked to structural loss in motor-related regions. This may reflect cumulative neurotoxicity from repeated withdrawals or seizures, or nutritional deficits such as thiamine deficiency, which particularly affect the motor cortex. The caudate’s involvement aligns with its role in habit formation; higher MATT scores may indicate a shift from goal-directed to compulsive, habitual alcohol use, consistent with striatal degeneration seen in both human and animal studies (78). These structural changes could underlie motor or behavioral rigidity in severe AUD.

### 4.4 PENN-brain correlation

The observed positive correlation between craving (PENN) and occipital volume should be interpreted cautiously, as it was no longer significant after controlling for age. Younger patients, who tend to have higher craving and larger brain volumes, may explain this association. Alternatively, individuals with preserved occipital cortex might be more visually cue-reactive to alcohol, though structural volume alone may not fully support this. Interestingly, lower craving in some patients could reflect more advanced neural damage and blunted reward processing, a pattern sometimes seen in chronic, cognitively impaired alcoholics. No link was found between craving and frontal volume, leaving open questions about inhibitory control. Future fMRI studies could clarify whether craving intensity relates to functional activity in occipital or frontal regions.

### 4.5 Mood symptoms and brain structure

Our findings suggest distinct neural correlates for depression and anxiety in AUD. Higher depression scores correlated with greater gray matter and amygdala volumes, possibly reflecting preserved emotional insight or a predisposition to heightened affect. In contrast, severe brain damage may blunt emotional awareness, leading to lower reported depression. The positive link between depression and angular gyrus volume may relate to default mode network activity and rumination, while reduced left frontal pole volume aligns with known associations between frontal deficits and depressive symptoms. Anxiety showed different patterns, emphasizing that mood symptoms in AUD engage unique, sometimes opposing, brain mechanisms.

Anxiety in AUD was negatively correlated with orbitofrontal and parietal volumes, consistent with impaired top-down regulation and stress-related neurotoxicity. Reduced orbitofrontal volume may weaken inhibition of limbic anxiety responses, while parietal atrophy (angular, supramarginal gyri) could reflect disrupted self-other processing or attentional control. Anxiety may also reflect withdrawal-related hyperexcitability in those with greater brain damage. Though correlated with depression, anxiety showed stronger links to structural deficits, suggesting it may be more directly neurobiological, whereas depression may arise from a more complex interplay of brain and psychological factors.

### 4.6 Integration and implications

Our findings reveal that alcohol dependence results in structural brain changes that mimic and exceed typical aging, particularly in regions critical for self-regulation and emotional processing such as the frontal cortex, insula, cingulate, and striatum. These neural alterations align with common clinical symptoms of AUD, including impaired impulse control, craving, and emotional instability. The marked brain age acceleration underscores that AUD is not solely a behavioral disorder but also a neurodegenerative condition with implications for long-term cognitive health. Individuals with AUD may have reduced cognitive reserve, raising their risk for early-onset dementia, including Alzheimer’s. Encouragingly, partial recovery of brain volume and cortical thickness is possible with prolonged abstinence, especially in white matter and select gray matter regions. However, some structural losses may be permanent. These insights support the importance of early intervention, continuous monitoring of cognitive function, and integrating brain health into AUD treatment planning. It is also important to note that although sex distribution was balanced between groups (two females in each), the very small number of women in the sample limits generalizability. Thus, our findings primarily reflect male patients with alcohol dependence, and future studies should include larger and more sex-balanced samples to clarify potential sex-specific neuroanatomical patterns.

### 4.7 Limitations

This study’s limitations include a modest sample size and the overall underrepresentation of females (only two in each group), which limits generalizability—especially given possible sex differences in alcohol-related brain atrophy. The cross-sectional design prevents causal inferences or tracking recovery. Although volBrain is validated, automated segmentation may misestimate certain regions, such as the hippocampus at 1.5T. Multiple comparisons were not fully corrected, raising the risk of Type I errors in exploratory correlations. Smoking, common in the alcohol group (80% *vs*. 15% in controls), and other potential confounds (e.g., hepatitis C, genetic factors) were not controlled. Although smoking status was recorded, it was not statistically controlled in our analyses due to sample size limitations. This imbalance may have contributed additively to the observed group differences in brain morphometry, and the presence of smokers in the control group may also help explain the relatively large mean absolute error in brain age estimates among controls. Given the established link between chronic smoking and increased brain-predicted age, future studies with larger samples should include smoking as a covariate when estimating neurobiological aging. The predictive accuracy of the brain age model was limited in our sample, with weak correlations and high error rates in both groups. In addition, regional brain-predicted age estimates are derived from fewer structural features than whole-brain age and are therefore more sensitive to scan parameter differences, which limits their reliability and suggests these results should be interpreted as exploratory. Future studies using models optimized for clinical populations are warranted. In addition, although several standardized effect sizes (Hedges’ g) appeared numerically large, the absolute differences corresponded to only millimeters in cortical thickness or a few cubic centimeters in volume, consistent with typical morphometric findings. This underscores the need to interpret standardized effects within the neuroimaging context rather than according to conventional behavioral science benchmarks. Finally, control participants were not entirely “super healthy,” which may introduce some variability, though major group differences remain robust.

### 4.8 Clinical implications

Neuroimaging can be a valuable tool in assessing alcohol-related brain damage, especially in mid-life individuals with chronic use. The concept of “brain age” may serve as a powerful motivator in treatment by making the impact of alcohol tangible. Identifying severe atrophy through MRI may guide the need for cognitive rehabilitation and closer monitoring of patients at risk for complications. Correlations between brain structure and clinical symptoms suggest tailored interventions: patients with high MATT and motor cortex loss may need fall-risk evaluation, while those with depression and frontal atrophy may benefit from neuroplasticity-enhancing therapies. Addressing anxiety is also critical, as orbitofrontal damage may contribute to relapse risk. A comprehensive, neuropsychologically informed treatment approach is essential.

## 5 Conclusion

This study demonstrates that alcohol dependence is associated with widespread brain structural deterioration, marked by both global and region-specific changes consistent with accelerated aging. Key brain alterations—particularly in frontal, sensory-motor, and striatal regions—correlate with addiction severity, craving, depression, and anxiety, underscoring their clinical relevance. These findings highlight alcohol addiction as both a psychiatric and neurodegenerative condition. Early intervention and sustained abstinence are critical to preventing further brain damage. Our results support the integration of cognitive screening, brain imaging, and mental health care into addiction treatment, and call for interdisciplinary approaches that address both the psychological and neurological dimensions of alcohol use disorder.

## Data Availability

The datasets supporting the findings of this study are openly available in the Figshare repository at https://doi.org/10.6084/m9.figshare.29488127.
